# “Getting every smoker to participate and quit - GEMPAQ V2.0”, a personalised smoking cessation smartphone app in Malaysia: A pilot randomised controlled trial

**DOI:** 10.1177/20552076261453665

**Published:** 2026-06-11

**Authors:** Ina Sharyn Kamaludin, Anne Yee, Lim Sin How, Mahmoud Danaee, Amer Siddiq Amer Nordin, Kan Wei Yeong, Zarwina Yusoff, Wong Yiik Sang, Farizah Mohd Hairi

**Affiliations:** 1 Department of Social and Preventive Medicine, Faculty of Medicine, 37447 Universiti Malaya, Kuala Lumpur, Malaysia; 2 Nicotine Addiction Research Collaborating Group, Universiti Malaya Centre for Addiction Science Studies, Universiti Malaya, Kuala Lumpur, Malaysia 37447; 3 Jeffrey Cheah School of Medicine and Health Sciences, Clinical School Johor Bahru, 175031 Monash University Malaysia, Johor Bahru, Malaysia; 4 Department of Psychological Medicine, Faculty of Medicine, 37447 Universiti Malaya, Kuala Lumpur, Malaysia; 5 Department of Software Engineering, Faculty of Computer Science and Information Technology, 37447 Universiti Malaya, Kuala Lumpur, Malaysia; 6 Department of Computer System and Technology, Faculty of Computer Science and Information Technology, Universiti Malaya, Kuala Lumpur, Malaysia 37447; 7 Faculty of Computer Science and Information Technology, 301405 University of Malaysia Sarawak, Sarawak, Malaysia

**Keywords:** smartphone app, smoking cessation, mHealth, pilot randomised controlled trials, personality traits, Malaysia

## Abstract

**Background:**

Despite rigorous tobacco control efforts over many years, smoking prevalence in Malaysia remains high. This study evaluates the effectiveness of a novel smartphone smoking cessation app, tailored to users’ five-factor personality traits, in increasing quit rates.

**Methods:**

A double-blind, parallel, two-arm randomised controlled pilot trial compared a personalised smoking cessation app (intervention) with a basic app (control). The online study, conducted across Malaysia with a 90-day follow-up, enrolled 152 adult smokers (N = 152).

**Results:**

Most participants were male (88.5%; mean age, 38.5 years [SD = 11.75]). In complete-case analysis (N = 87), the GEMPAQ V2.0 intervention group (*n* = 44) showed higher app utilisation than controls (*n* = 43; mean 117.63 vs. 95.25 sessions; adjusted *p* = 0.007) and marginally higher 7-day point-prevalence abstinence rates at 30 days (49.0% vs. 9.4%; aOR 1.02, 95% CI 1.00–1.05, *p* = 0.087), 60 days (65.2% vs. 16.3%; aOR 1.02, 95% CI 1.00–1.05, *p* = 0.100), and 90 days (59.1% vs. 25.6%; aOR 1.02, 95% CI 1.00–1.05, *p* = 0.088). mHealth app usability scores also favoured intervention in the “Ease of Use” dimension (*p* = 0.036) and “Interface and Satisfaction” dimension (*p* = 0.037). The conscientiousness trait showed the strongest significant correlations with GEMPAQ V2.0.

**Conclusion:**

Personalised GEMPAQ V2.0 users appeared to demonstrate promising indicators of utilisation, including the number of apps assessed and usability over time, and reported encouraging quit rates, particularly among smokers with a dominant conscientiousness personality trait.

## Introduction

Smartphone or mobile-based applications (apps) have revolutionised the landscape of smoking cessation interventions. They provide convenient access to smoking cessation assistance at any time and from anywhere. This innovative and cost-effective tool is essential in combating the biggest public health threat: tobacco smoking.^1^ In Malaysia, the current prevalence of tobacco smoking is 19.0%.^2^ Around 4.8 million Malaysians aged 15 years and above are current smokers, with almost 30,000 preventable deaths each year.^2–4^

Although there are numerous smoking cessation aids available, ranging from evidence-based recommendations (e.g., nicotine replacement therapies (NRT)) to globally affordable health-care interventions (e.g., brief advice from a health care provider, telephone helplines, printed self-help materials),^5^ smoking’s addictive nature,^6,7^ coupled with limited access to and adherence to effective treatments, poses major barriers.^8,9^ Technology can help overcome these obstacles, and the increasing popularity of smartphone usage has enabled mobile apps to offer more sophisticated tools for interaction with participants, including intuitive user interfaces and enhanced functionality.^10^ Thus, many smartphone apps have been developed to aid smoking cessation in the last decade.^11,12^ Related randomised controlled trials (RCTs) investigating apps for smoking cessation have shown mixed results, highlighting both negative and positive effects on smoking cessation rates.^13–21^ Systematic reviews indicated low efficacy rates for such app interventions due to insufficient evidence; however, ongoing studies will continue to refine the effectiveness of these interventions.^22–24^

Among the main drawbacks of these apps is that they are diverse in content and functionality.^22,23^ Additionally, the varied features do not suit the characteristics or personalities of smokers.^9,25,26^ The majority of the apps are generic, lacking scientific evidence, and are not tailored or personalised to meet the needs of each smoker; however, these factors can be beneficial.^27–29^ Global research has found that personality-based interventions enhance behaviour change.^9,30–32^

Ubiquitous smartphone use highlights the timeliness of app-based tobacco cessation interventions. Globally, users average 3 hours and 15 minutes of daily screen time, while Malaysians exceed this at 4 hours and 37 minutes.^33^ This extensive exposure positions apps as a viable platform for personalised smoking cessation interventions. Moreover, the International Tobacco Control (ITC) Malaysia 2020 national survey found that 85.2% of smokers intended to quit smoking in the next 6 months and that they were more likely to have used a mobile app to aid their smoking cessation efforts (aOR = 2.58; *p* = 0.001).^34^

This pilot RCT aimed to evaluate the feasibility of a large-scale trial to assess the usability and efficacy of GEMPAQ V2.0, a novel personalised bilingual (in English and Bahasa Malaysia, the national language) app among Malaysian adult smokers. The research team hypothesised that the use of a mobile app with modules tailored to the smoker’s personality traits would increase the cessation rate.

## Methods

### Trial design and setting

This double-blind, two-arm parallel pilot randomised controlled trial (RCT) was conducted online across Malaysia over 90 days (3 months) from January to September 2024. GEMPAQ V2.0, built on the React Native framework by the Nicotine Addiction Research Collaborating Group (NARCC) at the Universiti Malaya Centre for Addiction Science Studies (UMCAS), employed user interface design guided by Nielsen’s Theory of Usability,^35^ focusing on efficiency, satisfaction, and effectiveness, incorporating personalisation based on the Big Five Personality Traits.^36^ This model examines how tailoring module preferences to a smoker’s personality boosts engagement,^37,38^ aligning with recent recommendations for digital health interventions.^39,40^ At baseline, after participants completed the integrated personality traits questionnaire in the app, their traits were automatically calculated, enabling the release of tailored smoking cessation app modules based on their dominant personality traits. The preferred modules were derived from an algorithm based on results from an earlier feasibility mapping pre-post study of GEMPAQ V1.0, from which the present app design and methodology were adapted.^41^ For this pilot study, all participants were requested to use the GEMPAQ V2.0 app exclusively during the 90-day trial to aid their smoking cessation, without any other co-interventions (pharmacotherapy or smoking cessation programs).

### Ethics approval and trial registration

This study has been approved by the Medical Research Ethics Committee, Universiti Malaya Medical Centre (MREC ID#2022428-11196) and registered with the National Medical Research Registry of Malaysia (NMRR ID-22-02155-KUI) and in the International Standard Randomised Controlled Trial Number (ISRCTN) Registry (United Kingdom): ISRCTN91299538.

### Recruitment

Recruitment drives were undertaken extensively via various NARCC’s public health outreach activities, digital advertising and referrals for the past two years (2023 – 2024). Potential participants’ suitability was assessed by completing an online eligibility questionnaire, which included the patient information sheet. Eligible participants were then guided via email and/or messaging apps (WhatsApp/Telegram) to download the apps directly from the Google Play Store. For those unable to download independently, the corresponding Android application package (APK) was sent to them. Informed online consent built into the app was obtained prior to commencement of the study, and participants were made aware of the existence of the two treatment conditions. However, they did not know the specific app version allocated to them. Self-registration was done by creating their own login identity and password. Non-eligible participants would receive a ‘thank you’ study decline message.

### Participants

A total of 152 adult smokers in Malaysia were recruited online and equally randomised to two groups: intervention, *n* = 76, and control, *n* = 76.

### Inclusion criteria

Participants were eligible if they were aged 18 years or older, were current and active smokers (having smoked at least one combustible cigarette in the past 30 days and having a lifetime history of smoking at least 100 cigarettes)^42,43^ and had the intention to quit. Additional eligibility criteria included owning a smartphone (Android 12.0 or above), having internet access, providing online informed consent, expressing interest in quitting smoking within the next 14 days, and being able to communicate in either English or Bahasa Malaysia.

### Exclusion criteria

Participants who used alternative cessation treatments within the past 30 days, were registered in other smoking cessation programs, or used smartphones other than Android (e.g., iOS) were excluded.

### Randomisation

An independent project team member, uninvolved in data analysis or outcome evaluation, created the random allocation sequence via an online randomisation tool available at https://www.randomizer.org. Eligible participants were randomly allocated in a 1:1 ratio to either the intervention group (using a personalised app) or the control group (using a basic placebo app), without stratification or blocking. Allocation concealment was achieved through a centralised randomisation system, ensuring enrolling researchers had no access to the sequence before assignment. The trial used double-blinding: participants and outcome assessors remained unaware of group assignments. Blinding was preserved by providing coded versions of the app throughout the study.

### Intervention condition

**Figure 1.**
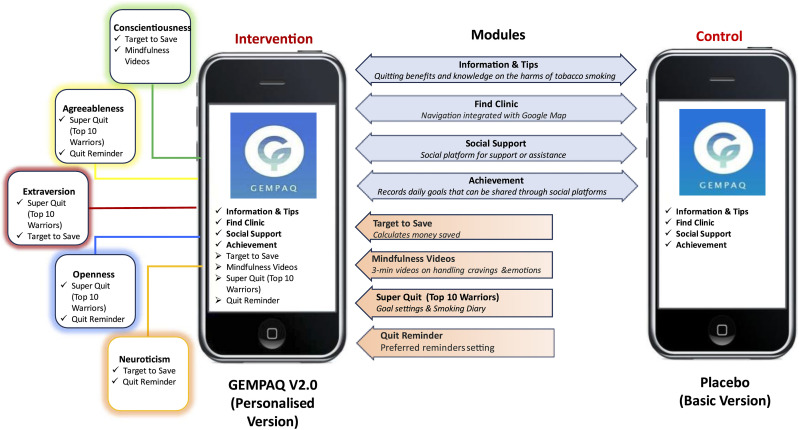
Intervention (Personalised Version) vs Control (Basic Version).

The intervention, which is the personalised and full version of the app, consists of eight modules, of which the four core modules, as described below, were also provided to the control group, as shown in Figure 1.

The four core modules: 1) “Information and Tips” module has details about quitting benefits and knowledge on the harms of tobacco smoking, 2) “Find Clinic” module, integrated with Google Maps, provides keen users with navigation capabilities to locate the nearest primary care clinics and community pharmacies for smoking cessation programs, 3) “Social Support” module allows participants to connect with family and friends through phone contacts and third-party channels like WhatsApp or Facebook and 4) “Achievement” module allows users to create daily goals and share them on various social media platforms.

The next four personalised modules: 5) “Target to Save” module allows users to calculate the potential savings from quitting smoking, 6) “Mindfulness Videos” module allows users to watch any of the four 3-minute smoking cessation videos from the playlist at their convenience, 7) “Super Quit” module, featuring the Top 10 Warriors and Smoking Diary, allows smokers to set targets as part of their quit smoking goals and 8) “Quit Reminder” module allows users to customise the time and intervals for reminders to quit smoking, enabling self-reminders at their preferred time.

### Control condition

Control participants were exposed to the placebo, which is the basic version of GEMPAQ V2.0, as shown in Figure 1 the same first four core modules as described in the intervention section above will be provided: 1) “Information and Tips”, 2) “Find Clinic”, 3) “Social Support” and 4) “Achievement”.

### Baseline and follow-up

Baseline questionnaire was self-administered via an in-app survey, which included sociodemographic, smoking status and personality traits (see Supplementary Figure 1). Personality was measured using the 20-item Mini International Personality Item Pool (Mini-IPIP), where participants rated brief statements on a 5-point Likert scale (1 = Very Inaccurate to 5 = Very Accurate).^44^ The Cronbach’s alphas for each of the personality dimensions are 0.77 (Agreeableness), 0.74 (Conscientiousness), 0.82 (Extraversion), 0.78 (Neuroticism) and 0.70 (Openness).^44^

Self-reported 7-day point prevalence abstinence (PPA) rates^45^ were assessed every 30 days. The overall usability of GEMPAQ 2.0 was evaluated by participants who completed the final 90-day survey using the mHealth App Usability Questionnaire (MAUQ), with an overall Cronbach’s alpha = 0.914.^46^ The 18-item MAUQ uses a 7-point Likert scale to assess the usability of mhealth apps. Participants rate their level of agreement with statements related to ease of use, interface and satisfaction, and usability of the app, with responses ranging from 1 (strongly disagree) to 7 (strongly agree).^46^ All surveys were conducted in both English and the national language, Bahasa Malaysia, using validated and translated Malay versions of the questionnaires.^47–49^ Upon completing the questionnaire at the end of every 30 days, participants received MYR 30.00 (approximately USD 7.05). Participants were provided MYR 90.00 (approximately USD 20.25) as maximum compensation for completing the entire study. Follow-up assessments were also administered via in-app surveys at the 30, 60 and 90-day time points. Non-responding participants were sent text messages every other day for a maximum of three times. After that, they were considered as nonabstainers, as per the intention-to-treat principle.

### Measures

#### Primary outcome

As usability is a prerequisite for the success of health and wellness mobile apps,^50^ this pilot trial aims to provide insights into the usability experience of GEMPAQ V2.0 by exploring the app utilisation and how users perceive key usability dimensions. Utilisation of the app was defined as the frequency with which participants opened and interacted with the specific app module. These passive objective usage metrics were captured in real time via the Google Firebase database. We examined the full 90-day frequency of app access (see Supplementary Figure 1 and Supplementary Table 1). The overall usability of GEMPAQ V2.0 was evaluated by participants who completed the final 90-day time point using MAUQ.^46^

### Secondary outcomes

To evaluate GEMPAQ V2.0’s effectiveness in supporting smoking cessation, we measured self-reported 7-day PPA rates at 30, 60, and 90-day time points, as recommended for smoking abstinence measures.^45^ After downloading the app, participants set a quit date, at which point the trial commences. An in-app survey was self-administered at every 30-day time point starting from the quit date and the 7-day PPA rates were reported.

The relationship between smokers’ personality traits according to the Big Five five-factor personality traits^36^ using the Mini-IPIP^44^ and its engagement with GEMPAQ V2.0 modules was also examined.

### Sample size

The sample size calculation was based on related studies evaluating app interventions among adult smokers.^14,16,51^ As the main focus of this pilot RCT is on the usability of GEMPAQ V2.0 in assisting adult smokers to reduce and quit smoking, the study’s sample size is powered to show differences in usability, operationalised as the number of times participants opened their assigned app. A priori sample size calculation was performed using the G*Power program, which showed that the minimum number of participants required in each group according to repeated measures analysis of variance with power (1-β) of .80, an effect size of f =.175, three measurements, and a significance level of α =.05, is 54.

While digital health studies offer substantial cost and time efficiencies, they often suffer high participant attrition rates of 20%–50%.^52,53^ Therefore, for this trial, 87 participants were recruited for complete case analysis (CCA) and 152 for intention-to-treat (ITT) analysis at the end of the trial (90 days). Despite an average 43% attrition rate across both arms, which is typical for digital health studies,^52,53^ the recruitment exceeded the recommended powered sample size calculation, ensuring robust statistical inference.

### Analyses

Analyses were computed using IBM SPSS® Statistics (Version 23.0) and JASP 0.19.1 software. Demographic characteristics and personality trait measures at baseline were compared between study groups using two-sample *t*-tests for continuous variables and Pearson’s ꭓ^2^ test for binary variables. As this study used automated electronic data collection, there were no missing values in the baseline data and other survey questionnaires; the app also includes a data integrity check to prevent users from entering invalid data (e.g., maximum age is 99).

Regression models were used to compare the two treatments on app utilisation, language used and smoking cessation outcomes. Analyses of follow-up data were restricted to the population of participants who completed and provided data at each time point. Generalised Estimating Equations (GEE) were used to account for repeated abstinence measures in the longitudinal dataset. This approach yielded robust estimates of the GEMPAQ V2.0 treatment effects while adjusting for baseline imbalances. The models used a logit link function and a Bernoulli distribution, with odds ratios (ORs) and 95% CIs reported as effect size measures. Sensitivity analyses included complete case analysis (CCA), which retained participants with observed outcomes at each follow-up time point under the Missing Completely at Random (MCAR) assumption. In addition, an intention-to-treat (ITT) analysis used the last observation carried forward (LOCF) method, under the assumption that all participants who dropped out remained smokers.^54^ Subsequently, abstinence rates by the most dominant personality trait were examined descriptively at the 30, 60 and 90-day time points.

Further analyses include examining the correlation of smokers’ personality traits and the usage of the various app modules using Spearman’s rho correlation coefficients. Ideal for initial exploration of app usage associations, Spearman’s rho, which captures monotonic relationships, is apt for assessing this correlation.^55^

Participants could withdraw from the study at any point, without having to justify their decision. The app was not modified during the trial. The study was conducted, and the results are presented in conformity with the 2010 Consolidated Standards of Reporting Trials (CONSORT): extension to randomised pilot and feasibility trials guidelines.^56^ There was no data-monitoring committee.

## Results

### Recruitment and baseline characteristics

**Figure 2.**
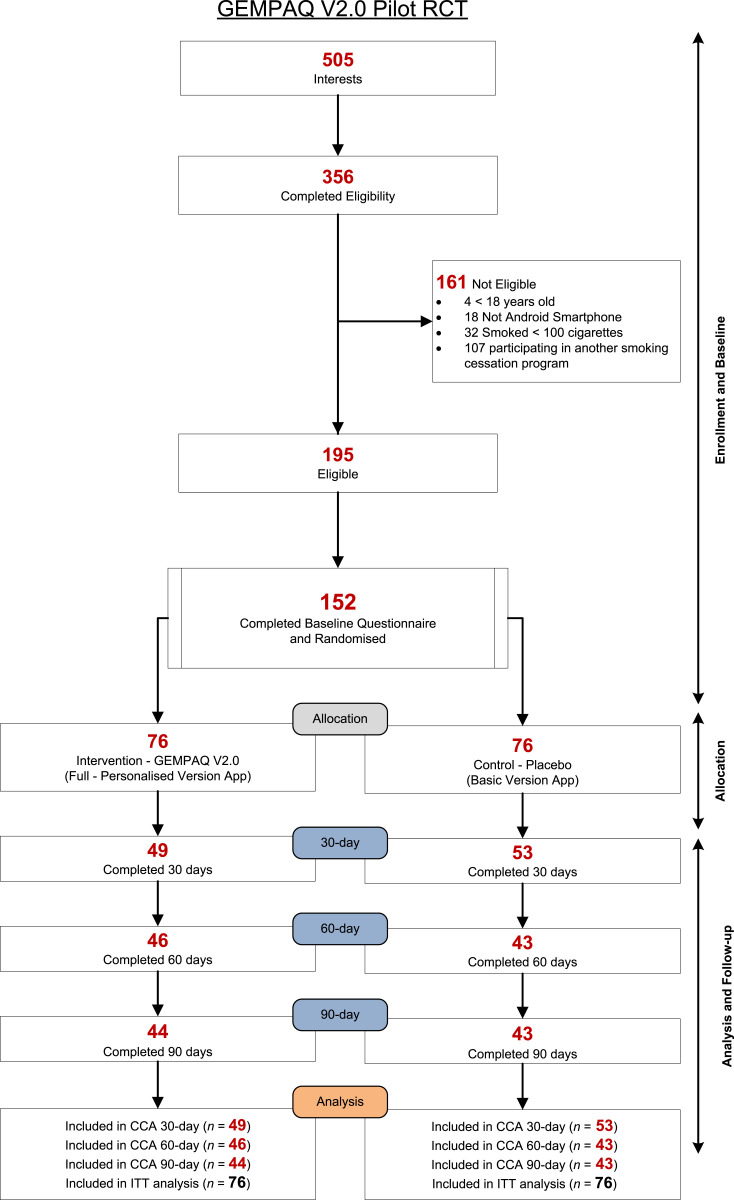
CONSORT flowchart of GEMPAQ V2.0 pilot RCT.

Initially, 505 individuals were interested, but only 356 completed eligibility and were assessed accordingly, of whom 161 participants were excluded, resulting in 195 eligible participants. Eventually, 152 participants completed the baseline and were randomised with equal probability (intervention, *n* = 76, control, *n* = 76). As anticipated for studies in digital health, attrition was 43%. Eventually, 87 daily smokers completed the study (intervention, *n* = 44, control, *n* = 43) and were included in the CCA. The differential of follow-up retention rates at the 90-day time point was not observed between the two groups (57.9% (44/76) vs. 56.6% (43/76), *p* = 1.000) (Figure 2).

At baseline, the participants’ mean (standard deviation (SD)) age was 38.5 (11.75) years. Most participants were male (88.5%, *n* = 77), of Malay ethnicity (52.7%, *n* = 46), with a mean (SD) daily cigarette consumption of 9.4 (5.52). Conscientiousness personality trait (46.0%, *n* = 40) appeared to be the most dominant personality trait. Apart from age, ethnicity, monthly income, age at which smoking started, and prior quit attempt effort, the GEMPAQ V2.0 intervention group and the control group appeared balanced in baseline characteristics (*p* ≥ 0.05) (Table 1).

**Table 1.** Baseline characteristics for GEMPAQ V2.0 pilot RCT.

Characteristics	Intervention group	Control group	Total	*p*-value
	(*n* = 44)	(*n* = 43)	(*n* = 87)	
	n (%)	n (%)	n (%)	
Sex				
Male	40 (90.9)	37 (86.0)	77 (88.5)	0.477
Female	4 (9.1)	6 (14.0)	10 (11.5)	​
Age (mean (SD)) years	35.3 (10.22)	41.6 (13.27)	38.5 (11.75)	0.014*
Ethnicity				
Malay	30 (68.2)	16 (37.2)	46 (52.7)	0.014*
Chinese	5 (11.4)	9 (20.9)	14 (16.2)	​
Indian	1 (2.3)	7 (16.3)	8 (9.3)	​
Others	8 (18.2)	11 (25.6)	19 (21.8)	​
Marital Status				
Married/Cohabitating	23 (52.3)	27 (62.8)	50 (57.5)	0.067
Separated/Divorced/Widowed	1 (2.3)	5 (11.6)	6 (6.9)	​
Single	20 (45.5)	11 (25.6)	31 (35.6)	​
Education				
Low (upper secondary and lower)	22 (50.0)	22 (51.2)	44 (50.6)	0.919
Moderate (diploma certificate)	7 (15.9)	8 (13.3)	15 (17.2)	​
High (university and higher)	15 (34.1)	13 (30.2)	28 (32.2)	​
Employment Status				
Full Time	32 (72.7)	36 (83.7)	68 (78.2)	0.414
Part Time	2 (4.5)	3 (7.0)	5 (5.7)	​
Unemployed/Retired/Home Duties/Others	7 (15.9)	3 (7.0)	10 (11.5)	​
Student	3 (6.8)	1 (2.3)	4 (4.6)	​
Monthly Income				
Low (less than USD1088)	37 (84.1)	23 (53.5)	60 (69.0)	0.003*
Middle (USD1089 to USD2461)	4 (9.1)	16 (37.2)	20 (23.0)	​
High (USD2462 and above)	3 (6.8)	4 (9.3)	7 (8.0)	​
Personality Trait - Most Dominant				
Agreeableness	7 (15.9)	5 (11.6)	12 (13.8)	0.757
Conscientiousness	19 (43.2)	21 (48.8)	40 (46.0)	0.669
Extraversion	3 (6.8)	3 (7.0)	6 (6.9)	1.000
Neuroticism	8 (18.2)	7 (16.3)	15 (17.2)	1.000
Openness	7 (15.9)	7 (16.3)	14 (16.1)	1.000
Smoking Behaviour				
Age started smoking years (mean (SD))	18.3 (3.26)	17.1 (2.51)	17.7 (2.89)	0.049*
Cigarettes per day (mean (SD))	8.5 (6.08)	10.2 (4.96)	9.4 (5.52)	0.150
At least one prior quit attempt	29 (65.9)	17 (39.5)	46 (52.9)	0.018*

*p < 0.05.

### Primary outcome

#### GEMPAQ V2.0 usability

For CCA, the GEMPAQ V2.0 intervention group (*n* = 44) demonstrated significantly higher app utilisation frequency than the control group (*n* = 43), with a mean of 117.63 (SD = 155.80) sessions versus 95.25 (SD = 74.62); adjusted linear regression *p* = 0.007, controlling for 7-day PPA and age. On the MAUQ dimensions,^46^ Welch’s t-tests revealed modest intervention advantages in the “Ease of Use” dimension with a mean of 6.00 (SD = 1.01) vs. 5.67 (SD = 0.09); *p* = 0.036 and “Interface and Satisfaction” dimension with a mean of 6.02 (SD = 1.07) vs. 5.58 (SD = 0.85); *p* = 0.037, but not in the “Usability” dimension 6.11 (SD = 0.87) vs. 6.09 (SD = 0.97); *p* = 0.920. Language use further highlighted cultural relevance: 77.3% (34/44) of intervention participants used Bahasa Malaysia (national language) compared to 58.1% (25/43) in controls (*p* = 0.033), while English use was lower at 22.7% versus 41.9% (Table 2).

**Table 2.** Comparison of GEMPAQ V2.0 usability between intervention and control groups at 90-day time point.

​	Intervention group *(n* = 44)	Control group *(n* = 43)	*p*-value
*Utilisation of assigned app* ^a^ *, mean (SD)*			
Frequency of app assessed	117.63 (155.80)	95.25 (74.62)	0.007*
*App Usability, mean (SD)*			
Ease of Use^†^	6.00 (1.01)	5.67 (0.09)	0.036*
Interface and Satisfaction^†^	6.02 (1.07)	5.58 (0.85)	0.037*
Usability^†^	6.11 (0.87)	6.09 (0.97)	0.920
*Language Used* ^a^ *, n (%)*			
Bahasa Malaysia (national)	34 (77.3)	25 (58.1)	0.033*
English	10 (22.7)	18 (41.9)	​

^a^Two-sided *p*-values calculated from Regression Models adjusted for 7-day PPA abstinence and age. Unadjusted two-sided *p*-values were very similar. *p < 0.05.

^†^Dimensions from mHealth App Usability Questionnaire (MAUQ) - Scale of 1 to 7: strongly disagree to strongly agree. Two-sided Welch’s t-tests were used to compare means between groups. *p < 0.05.

The frequency of accessing GEMPAQ V2.0 modules for CCA (intervention, *n* = 44, control, *n* = 43) was examined, which confirmed that participants actively used the apps (see Supplementary Table 1). Super Quit (55.4%) is the most frequently accessed module by participants in the intervention group, followed by Target to Save (13.8%) and Information & Tips (8.6%). As only four core modules are available in the control group, the highest accessed module is Information & Tips (58.5%), followed by Find Clinic (18.1%) and Social Support (13.7%).

Usability data were collected from participants completing the 90-day follow-up. MAUQ’s three dimensions were measured: 1) Ease of Use, 2) Interface and Satisfaction, and 3) Usability^46^ for this study to determine the utilisation of GEMPAQ V2.0 (see Supplementary Table 2). Both the intervention group (*n* = 44) and control group (*n* = 43) demonstrated high app usability, and most individual items showed no statistically significant differences (*p* > 0.05, two-sided tests), indicating comparable perceived app usefulness across groups. Only three items reached significance (*p* < 0.05): 1) “Ease of Use” dimension: item “The app’s interface allowed me to use all the functions offered by the app”, the intervention group reported a mean score of 5.86 (SD = 1.05), while the control group had a mean of 5.42 (SD = 0.85), with a statistically significant difference (*p* = 0.035). This suggests that participants in the intervention group felt more confident in utilising the app’s functions compared to those in the control group.

In the dimension of “Interface and Satisfaction”, 2) “The app adequately acknowledged and provided information to let me know the progress of my action”, the intervention group scored a higher mean score of 5.95 (SD = 0.96) compared to 5.44 (SD = 0.96) for the control group (*p* = 0.015). This indicates that participants in the intervention group felt more informed about their progress when using the app, suggesting that the intervention may have included enhanced features for feedback and progress tracking. The other significant difference was found regarding 3) “The amount of time involved in using this app has been fitting for me”, with the intervention group reporting a mean score of 6.07 (SD = 1.02) compared to 5.44 (SD = 0.91) for the control group (*p* = 0.003).

Overall, significant differences emerged in the “Ease of Use” dimension, favoring the intervention group (*p* = 0.036), and the “Interface and Satisfaction” dimension (*p* = 0.037). These findings indicate that intervention participants perceived the app as easier to navigate and more satisfactory overall. In contrast, overall “Usability” dimension scores showed no significant difference (*p* = 0.920), reflecting comparably high acceptability of GEMPAQ V2.0 in both groups (Table 2).

### Secondary outcomes

#### Smoking abstinence

For the CCA analysis, the Time*Group interaction using GEE revealed an overall group difference in 7-day PPA over time, with abstinence rates rising in the intervention group (24/49 [49.0%] at 30 days, 30/46 [65.2%] at 60 days, and 26/44 [59.1%] at 90 days) but remained lower in the control group (5/53 [9.4%], 7/43 [16.3%], and 11/43 [25.6%]), respectively. Adjusted odds ratios (aOR^††^; time + group + time*group + ethnicity + age) were consistently 1.02 (95% CI: 1.00-1.05) across time points at 30 days (*p* = 0.087); 60 days (*p* = 0.100) and 90 days (*p* = 0.088) (Table 3 and Supplementary Figure 2).

**Table 3.** Summary of self-reported 7-day PPA.

Self-reported 7-day PPA^1^	Time points	Intervention		Control		Difference from control^2^	
		N	*n* (%)	N	*n* (%)	aOR^††^ (95% CI) ethnicity + age	*p*-value
CCA	30-day	49	24 (49.0)	53	5 (9.4)	1.02 (1.00-1.05)	0.087
	60-day	46	30 (65.2)	43	7 (16.3)	1.02 (1.00-1.05)	0.100
	90-day	44	26 (59.1)	43	11 (25.6)	1.02 (1.00-1.05)	0.088
ITT	30-day	76	24 (31.6)	76	5 (6.6)	1.01 (0.98-1.04)	0.418
	60-day	76	29 (38.2)	76	7 (9.2)	1.01 (0.99-1.04)	0.359
	90-day	76	26 (34.2)	76	11 (14.5)	1.01 (0.99-1.04)	0.355

aOR, adjusted odds ratio; CI, confidence intervals.

^1^Measured by a self-report of continuous abstinence from smoking in the previous 7 consecutive days.

^2^Time*Group Interactions using Generalized Estimating Equations Analysis with Control Group as reference.

^††^aOR Time + Group + Time*Group + Ethnicity + Age.

ITT analysis (intervention, *n* = 76, control, *n* = 76), shows diluted effects: intervention group (24/76 [31.6%] at 30 days, 29/76 [38.2%] at 60 days, 26/76 [34.2%] at 90 days); control group (5/76 [6.6%], 7/76 [9.2%], 11/76 [14.5%] correspondingly). GEE modelling produced smaller aORs^††^ of 1.01 (95% CI: 0.98-1.04 at 30 days; 0.99-1.04 at 60 and 90 days; all *p* > 0.35) (Table 3 and Supplementary Figure 2).

To account for baseline imbalances and improve precision, we performed adjusted analyses using GEE, incorporating established prognostic covariates^57–60^: ethnicity, age, monthly income, age at smoking initiation, and prior quit attempts. These results are presented in Supplementary Table 3. For CCA , unadjusted odds ratios trended towards significance at 30 days (OR 6.06, 95% CI 0.73–50.18, *p* = 0.095) and 60 days (OR 6.34, 95% CI 0.79–50.80, *p* = 0.082) but were not significant at 90 days (OR 2.80, 95% CI 0.36–21.12, *p* = 0.327). After adjusting for ethnicity, effects became significant: aOR^†^ 3.41 (95% CI 1.17–9.94, *p* = 0.025) at 30 days, 3.38 (95% CI 1.13–10.09, *p* = 0.029) at 60 days, and 3.72 (95% CI 1.24–11.10, *p* = 0.019) at 90 days, revealing sustained intervention benefits across all time points. (see Supplementary Table 3).

In examining the most dominant personality trait in the repeated self-reported 7-day PPA at 30, 60, and 90-day time points (Table 4), conscientiousness emerges as the most frequent dominant trait across all time points and groups. As conscientiousness consistently ranked highest, it may predict sustained abstinence in both intervention and control groups, potentially serving as a key moderator of mHealth efficacy. Neuroticism is prominent in the intervention group early on but fades, while low neuroticism in the control group abstainers is notable (0% at 30 and 60-day time points).

**Table 4.** 7-Day PPA by most dominant personality trait at 30, 60 and 90-day time points.

​	30-day		60-day		90-day	
	IG (*n* = 49) n (%)	CG (*n* = 53) n (%)	IG (*n* = 46) n (%)	CG (*n* = 43) n (%)	IG (*n* = 44) n (%)	CG (*n* = 43) n (%)
Abstinence						
7-day PPA	24 (49.0)	5 (9.4)	30 (65.2)	7 (16.3)	26 (59.1)	11 (25.6)
Most Dominant Personality Trait						
Agreeableness	4 (16.7)	1 (20.0)	5 (16.7)	1 (14.3)	4 (15.4)	1 (9.1)
Conscientiousness	7 (29.2)	3 (60.0)	11 (36.7)	4 (57.1)	10 (38.5)	5 (45.5)
Extraversion	2 (8.3)	0 (0.0)	2 (6.7)	1 (14.3)	2 (7.7)	2 (18.2)
Neuroticism	7 (29.2)	0 (0.0)	8 (26.7)	0 (0.0)	6 (23.1)	1 (9.1)
Openness	4 (16.7)	1 (20.0)	4 (13.3)	1 (14.3)	4 (15.4)	2 (18.2)

IG: Intervention Group, CG: Control Group.

### Personality traits and GEMPAQ V2.0 modules usage

To understand the role of GEMPAQ V2.0 in supporting smoking cessation based on each of the Big Five personality traits,^36^ Spearman’s rho correlation coefficients were used to examine the relationship and strength between smokers’ personality traits and their interaction with various GEMPAQ V2.0 modules, using the full 90-day frequency of app access for CCA (intervention, *n* = 44; control, *n* = 43). This approach enabled the assessment of how individual personality differences influenced app usage and, consequently, the quit effort (Table 5 and Table 6).

**Table 5.** Spearman’s rho correlation coefficients between personality traits and GEMPAQ V2.0 modules usage for intervention group (*n* = 44).

Variable	Agreeableness	Conscientiousness	Extraversion	Neuroticism	Openness
Achievement	0.353*	0.524***	-0.115	0.122	0.045
Find Clinic	0.246	0.559***	-0.089	-0.015	0.233
Information & Tips	0.174	0.600***	-0.065	0.047	0.330*
Social Support	0.078	0.341*	-0.078	-0.028	0.148
Mindfulness Videos	0.150	0.410**	0.007	0.074	0.084
Target to Save	0.139	0.539***	0.079	-0.009	-0.024
Quit Reminder	0.121	0.131	-0.216	0.312*	0.068
Super Quit	0.394**	0.466**	0.098	0.128	0.183

*p < .05, ** *p* < .01, *** *p* < .001.

**Table 6.** Spearman’s rho correlation coefficients between personality traits and GEMPAQ V2.0 modules usage for control group (*n* = 43).

Variable	Agreeableness	Conscientiousness	Extraversion	Neuroticism	Openness
Achievement	0.016	0.012	0.135	-0.027	0.089
Find Clinic	-0.143	-0.058	-0.005	0.014	0.274
Information & Tips	0.101	-0.011	0.254	-0.140	0.338*
Social Support	0.059	0.028	0.228	-0.218	0.118

*p < .05, ** *p* < .01, *** *p* < .001.

For the CCA intervention group (*n* = 44) at 90-day time point, the analysis indicated that certain personality traits, particularly conscientiousness and agreeableness, have significant positive correlations (*p <.05, **p <.01, ***p <.001) with various GEMPAQ V2.0 modules, suggesting that these traits may play a crucial role in the effectiveness of interventions aimed at achieving personal goals and improving well-being among participants. Conscientiousness appears to have significantly stronger correlations than agreeableness (Table 5).

Conscientiousness shows strong and consistent positive correlations with nearly all module usages, with the highest coefficients seen for “Information and Tips” (ρ = 0.600, ***p < .001), “Find Clinic” (ρ = 0.559, ***p < .001), “Target to Save” (ρ = 0.539, ***p < .001), and “Achievement” (ρ = 0.524, ***p < .001). These results suggest that individuals with a dominant conscientiousness trait are more likely to engage across these modules, especially those involving planning, achievement, and information gathering.

Agreeableness is moderately positively correlated with “Super Quit” (ρ = 0.394, **p < .01) and “Achievement” (ρ = 0.353, *p < .05), indicating individuals with high agreeableness are more likely to use these modules. These relate to positive goal tracking and major quitting milestones. Openness only shows a significant correlation with “Information and Tips” (ρ = 0.330, *p < .05), implying participants high in openness may prefer information-based features.

Neuroticism has a significant positive correlation only with “Quit Reminder” (ρ = 0.312, *p < .05), suggesting those higher in neuroticism might benefit from or use reminders more, possibly as a compensatory strategy for impulsivity or forgetfulness. Extraversion does not present any significant correlations with any module usage, with coefficients generally near zero or slightly negative, suggesting extraversion is not a key driver of usage for the intervention modules.

The analysis of the CCA control group (*n* = 43) at the 90-day time point indicated that personality traits generally exhibit weaker correlations with the GEMPAQ V2.0 reduced version modules than those in the intervention group for the corresponding modules (Table 6). Openness is the only trait showing a statistically significant correlation. Specifically, openness is positively correlated with “Information and Tips” usage (ρ = 0.338, *p < .05). This suggests that participants in the control group with higher openness are more likely to engage with modules focused on information and tips. Openness emerges consistently, also seen in the intervention group, as a driver for engaging with informational modules. This is likely due to these individuals’ general enthusiasm for exploring new ideas. All other traits (agreeableness, conscientiousness, extraversion, neuroticism) do not show significant correlations with any module usage, as all coefficients are small and nonsignificant. The overall lack of significant correlations across most personality traits and modules suggests that, without the intervention, module usage is less likely to be influenced by personality factors.

## Discussion

The GEMPAQ V2.0 intervention group showed greater app utilisation and a stronger preference for Bahasa Malaysia than the control group, underscoring the value of localised design for Malaysian users. Similar to other mHealth studies,^16,51,61^ higher app utilisation fosters greater adherence to smoking cessation interventions by enabling consistent access to tailored support, reminders, and behavioural cues, which together enhance quit rates. In contrast, standard smoking cessation services in Malaysia, primarily through clinic and Quitline programs, yield quit rates of 15–17% to about 25% at 6–12 months (180–360 days) among those who set a quit date.^62^ By comparison, this pilot trial showed the personalised app group outperforming controls, with 7-day PPA rates of 49.0% at 30 days, 65.2% at 60 days, and 59.1% at 90 days. Overall, these results indicate that tailoring apps to users’ personality traits may substantially improve quit success. This finding is consistent with another study using a personalised app intervention, which reported a 7-day PPA rate of 51.5% at 8 weeks (60 days) for CCA.^9^ In contrast, a recent meta-analysis of smartphone apps found more modest effects, with quit rates typically in the 10-20% range for 7-day PPA at 90 days or less, depending on app features, adherence, and population characteristics.^63^ Higher rates are often seen in short-term or feasibility studies and among engaged users, while longer-term and ITT analyses show more modest but still meaningful effects.^23,24,63^

Apart from having the intention to quit,^60,64^ GEMPAQ V2.0 likely enhanced early abstinence rates in the intervention group through personalised features and delivery in the national language, Bahasa Malaysia, alongside English. Ethnicity adjustment reinforced these effects (aOR^†^ 3.41-3.72, *p* < 0.05), indicating cultural tailoring amplified efficacy in this Malaysian cohort, where Malay ethnicity, prevalent among smokers^57^ per baseline data, predominated. As smoking cessation apps are adopted mostly by younger smokers^16,60,65^ and despite the nearly 6-year mean age difference between intervention and control groups which could potentially influence both smoking patterns and app engagement, no significant interactions emerged in sensitivity analyses adjusting for age. In this respect, age imbalance did not confound primary Time*Group effects, consistent with robust app efficacy across adult age strata. The pilot findings highlight engagement and cultural adaptations as key drivers of digital health efficacy.

In the CCA, the unadjusted intervention effect appeared to diminish between 60 and 90 days, a pattern consistent with cessation fatigue.^66^ This attenuation may reflect ‘app fatigue’ and waning novelty in the absence of ongoing support, although this mechanism cannot be confirmed in the present study.^67^ Future full-scale RCTs could target these endpoints in a larger population. The high attrition experienced could also undermine causal inferences and generalisability, as dropouts may differ systematically by Big Five personality traits, such as conscientiousness, since those who remain are often healthier or more motivated individuals.^38^ Real-world deployment feasibility would be better informed by a clearer description of attrition rates and patterns (including any between-group differences), especially given that mitigation strategies such as ITT analyses were applied. In the context of personality-tailored apps, these attrition-related findings delineate important boundary conditions for effectiveness and underscore the need for design refinements.^37^

The adoption, usability and general experience of mobile apps by end users are critical as they directly impact user retention and adherence to cessation programs.^68^ This is vital as research indicates that many users abandon mHealth apps due to poor usability, such as complex interfaces or a lack of engaging content.^46,50^ MAUQ scores for GEMPAQ V2.0 showed no significant differences by intervention group, indicating broad acceptability of the app’s core usability features. Both groups had positive perceptions regarding the “Usability” dimension, whereas significant differences were observed in the “Ease of Use” and “Interface and Satisfaction” dimensions, favoring the intervention group. Key areas, such as progress acknowledgment and perceived appropriateness of time spent using the app, indicated meaningful improvement for users in the intervention group. These findings suggest that while users generally appreciated various aspects of both app versions, tailored modules through the intervention positively influenced users’ perceptions of progress feedback and time investment, potentially leading to greater user satisfaction and a greater likelihood of continued use. For future app development, these findings indicate that a smooth, visually attractive interface is crucial, as it greatly impacts users’ motivation and ease of navigation, which are key factors in assessing and enhancing mHealth interventions.^49^ Furthermore, enhancements that integrate specific app modules, such as gamification features or social accountability components, can augment user retention.^69,70^ However, maintaining long-term engagement remains a challenge, and future app designs should focus on sustaining motivation beyond initial novelty effects.

The findings from this trial also identified personality traits, particularly conscientiousness, which showed a strong positive correlation with various GEMPAQ V2.0 modules used in the intervention group. Participants in the CCA intervention group (using the full app version) with dominant conscientiousness reported higher abstinence rates compared to those in the control group (using the reduced app version). Conscientiousness also showed the strongest association with the usage of GEMPAQ V2.0 modules among intervention group participants. These findings indicate that this personality trait likely plays a key role in shaping user behaviour and engagement in the app context. This result is consistent with earlier studies that have shown the conscientiousness trait is linked to quitting smoking^71^ and appears to protect against smoking advancement and persistence.^38^ In summary, the conscientiousness trait plays a key role in successful smoking cessation due to increased self-regulation, adherence to treatment and perseverance, making it an important factor to consider when designing personalised cessation interventions tailored to individuals’ personality profiles.^72,73^ From the other module usage results, the “Information and Tips” module was also frequently used by smokers with a high openness trait, underscoring their possible significance in seeking services and information. On the other hand, smokers with high neuroticism trait may react more strongly to the “Quit Reminder” module; however, they appeared not to favour the “Social Support” module. These findings highlight smokers’ preferences by personality traits and can guide app implementation for smoking cessation, including personalisation of strategies to individual profiles. In line with other studies,^39,40,74^ the use of a validated personalisation algorithm could be extended to app-based interventions targeting other critical health behaviours such as alcohol use and physical inactivity, thereby advancing mHealth apps and contributing to improved public health outcomes.

With the rising demand for efficient, personalised healthcare solutions, personality-tailored digital health interventions such as apps in aiding smoking cessation offer scalable mHealth tools for upper-middle-income countries (UMICs) like Malaysia, where near-universal mobile penetration,^75^ contrasts with public health strains from rapid urbanisation, diverse demographics, and escalating non-communicable diseases.^76^ These apps deliver on-demand, adaptive support customised to users’ behavioural patterns with multilingual support, and culturally attuned content to engage urban professionals and rural communities alike. Strengthening participation in tobacco cessation programs^7^ through personalised tools and real-time feedback can prevent relapse after quitting, improve quit rates, reduce tobacco-related inequities, and advance global health equity goals.^77,78^

### Strengths

This pilot RCT was fully automated, relying solely on smartphone capabilities without any human intervention. Online delivery further reduced costs and minimised labour demands compared to traditional methods. The dual-language GEMPAQ V2.0 app, available for free download from the Google Play Store, integrated all surveys, which were automatically released according to each participant’s predetermined schedule from their start of quit date. The trial’s double-blind design decreased bias, made intention-to-treat analysis possible, enhanced generalisability, and produced high-quality evidence of a causal relationship.

### Limitations

Several limitations should be acknowledged. First, there is the possibility that participants can use two or more devices, or that participants who know each other might know about the other sub-app. Although measures have been taken to minimise these, we cannot eliminate such possibilities. Secondly, the reliance on self-reporting smoking status is a major limitation. Studies have found that differences between self-reported smoking abstinence and biochemically verified abstinence can be substantial, often exceeding 40%.^79,80^ Consequently, care must be taken when interpreting outcomes of self-reported abstinence. Thirdly, this pilot RCT was conducted for 90 days, which might have been insufficient to determine precisely whether participants could achieve smoking cessation for a longer period. Thus, future trials over a longer study period are warranted to verify the long-term efficacy of GEMPAQ V2.0. Another drawback is the high attrition rate, which threatens the internal validity and could lead to selection bias.^81^ Therefore, before wider implementation, larger, longer-duration investigations are required. As only combustible cigarette smoking was used in this trial, further examination of types of tobacco/nicotine products (cigarettes, heated tobacco, e-cigarettes, etc.) could be included for future exploration. Finally, due to study fund limitations and the fact that Android holds 70.26% of the mobile operating system market in Malaysia,^82^ as well as being the leading mobile operating system worldwide,^83^ led to GEMPAQ V2.0 being built only on the Android platform, restricting smokers using iOS and other smartphone operating systems from participating in this study.

## Conclusion

Users of the personalised GEMPAQ V2.0 showed promising utilisation patterns, in terms of the number of apps assessed, sustained usability, and encouraging quit rates. This pilot study offers initial insights into utilisation patterns, personality traits’ influence, and adaptation preferences, particularly higher cessation success among smokers with a dominant conscientiousness personality trait. Future research should incorporate biochemical verification via wearable monitoring in larger trials with longer follow-up to address attrition and improve outcome reliability. The small sample size underscores the need for a full-scale efficacy RCT to advance personalised mHealth apps for smoking cessation.

## Supplemental material

Supplemental material - “Getting every smoker to participate and quit - GEMPAQ V2.0”, a personalised smoking cessation smartphone app in Malaysia: A pilot randomised controlled trial Supplemental material for “Getting every smoker to participate and quit - GEMPAQ V2.0”, a personalised smoking cessation smartphone app in Malaysia: A pilot randomised controlled trial by Ina Sharyn Kamaludin, Anne Yee, Lim Sin How, Mahmoud Danaee, Amer Siddiq Amer Nordin, Kan Wei Yeong, Zarwina Yusoff, Wong Yiik Sang and Farizah Mohd Hairi in Digital Health.

## Data Availability

Data obtained through this study may be provided to qualified researchers with an academic interest in tobacco control. Data or samples shared will be coded, with no PHI included. Approval of the request and execution of all applicable agreements (i.e., a material transfer agreement) are prerequisites to the sharing of data with the requesting party. The datasets generated during and/or analyzed during the current study are/will be available upon request from the Principal Investigator: Associate Professor Dr. Anne Yee. Email: anne.yee@monash.edu.*
