# Supplementary material for: “Getting every smoker to participate and quit - GEMPAQ V2.0”, a personalised smoking cessation smartphone app in Malaysia: A pilot randomised controlled trial
Source: Digit Health. 2026 Jun 11;12:20552076261453665. doi: 10.1177/20552076261453665 (PMC13261050; doi:10.1177/20552076261453665)
Supplement: Supplemental material - “Getting every smoker to participate and quit - GEMPAQ V2.0”, a personalised smoking cessation smartphone app in Malaysia: A pilot randomised controlled trial [file sj-pdf-1-dhj-10.1177_20552076261453665.pdf]

Supplementary Figure 1. Study design and details on when measures were collected

fx2

Supplementary Table 1. GEMPAQ V2.0 Modules 90-day Time Point Frequency of Access*

<!--Col Count:3-->	Intervention Group (*n* = 44)<!--Para Run-on-->	Control Group (*n* = 43)<!--Para Run-on-->
	n (%)	n (%)
Modules		
Achievement	57 (6.1)	37 (9.7)
Find Clinic	47 (5.0)	69 (18.1)
Information & Tips	81 (8.6)	223 (58.5)
Social Support	36 (3.8)	52 (13.7)
Mindfulness Videos	55 (5.8)	NA[1]
Target to Save	130 (13.8)	NA[1]
Quit Reminder	14 (1.5)	NA[1]
Super Quit	521 (55.4)	NA[1]
*Via passive usage data recording (automatic)		
[1]NA: Not available in basic app version		

Supplementary Table 2. MAUQ Descriptive Quantitative Analysis of GEMPAQ V2.0 Usability at 90-day Time Point

<!--Col Count:7-->	Intervention Group		Control Group		
Items*	Mean (*n* = 44)	SD	Mean (*n* = 43)	SD	*p*-value <!--Soft-enter Run-on-->(two-sided)
**Ease of Use**					
The app was easy to use	6.07	0.93	6.07	1.01	1.000
It was easy for me to learn to use the app	6.09	1.12	5.79	0.89	0.170
The navigation was consistent when moving between screens	6.02	0.90	5.70	0.89	0.099
**The app's interface allowed me to use all the functions offered by the app**	5.86	1.05	5.42	0.85	**0.035**
Whenever I made a mistake using the app, I could quickly recover	5.82	1.33	5.56	0.83	0.279
**Interface and Satisfaction**					
I like the interface of the app	5.59	1.47	5.42	0.98	0.528
The information in the app was well organized, so I could easily find <!--Soft-enter Run-on-->the information I needed	5.82	1.11	5.60	1.03	0.339
**The app adequately acknowledged and provided information to let me know the progress of my action**	5.95	0.96	5.44	0.96	**0.015**
I feel comfortable using this app in social settings	5.80	1.30	5.72	1.05	0.754
**The amount of time involved in using this app has been fitting for me**	6.07	1.02	5.44	0.91	**0.003**
I would use this app again	6.07	0.93	5.70	1.01	0.079

<!--Col Count:7-->	Intervention Group		Control Group		
Overall, I am satisfied with this app	5.98	1.00	5.74	0.95	0.255
**Usability**					
The app would be useful for my health and well-being	6.05	0.99	6.02	0.94	0.885
The app improved my access to healthcare services	6.07	0.95	6.14	1.06	0.746
The app helped me manage my health effectively	6.11	0.92	5.72	0.93	0.053
This app has all the functions and capabilities I expected it to have	6.05	1.03	5.88	0.96	0.428
I could use the app even when the Internet connection was poor or unavailable	5.86	1.09	6.09	1.19	0.350
This mHealth app provided an acceptable way to receive health care services, such as accessing educational materials, tracking my own activities, and performing self-assessments	6.00	0.94	6.21	1.08	0.336
* Scale of 1 to 7: strongly disagree to strongly agree					

Supplementary Figure 2. Self-Reported 7-day PPA

fx3 fx4

(a) (b)

Self-reported smoking abstinence at 30, 60 and 90-day time points for 7-day Point Prevalence Abstinence (PPA): (a) CCA; (b) ITT.

Supplementary Table 3. Summary of Self-Reported 7-day PPA According to Prognostic Covariates

<!--Col Count:19-->		Intervention		Control		Difference from Control[2]											
<!--Soft-enter replaced as Paramark--> 7-day PPA[1]	Time <!--Soft-enter replaced as Paramark--> Points	N	*n* <!--Soft-enter Run-on-->(%)	N	*n* <!--Soft-enter Run-on-->(%)	OR <!--Soft-enter Run-on-->(95% CI)	*p*-value	aOR[†] <!--Soft-enter replaced as Paramark--> (95% CI)<!--Soft-enter replaced as Paramark--> Ethnicity	*p*-value	aOR[b] <!--Soft-enter replaced as Paramark--> (95% CI)<!--Soft-enter replaced as Paramark--> Age	*p*-value	aOR[c] <!--Soft-enter replaced as Paramark--> (95% CI)<!--Soft-enter replaced as Paramark--> Monthly Income	*p*-value	aOR[d] <!--Soft-enter replaced as Paramark--> (95% CI)<!--Soft-enter replaced as Paramark--> Age Started Smoking	*p*-value	aOR[e] <!--Soft-enter replaced as Paramark--> (95% CI)<!--Soft-enter replaced as Paramark--> Quit Attempt	*p*-value
**CCA**	30-day	49	24 <!--Soft-enter Run-on-->(49.0)	53	5 <!--Soft-enter Run-on--	6.06 <!--Soft-enter Run-on-->(0.73-	0.095	3.41 <!--Soft-enter Run-on-->(1.17-9.94)	**0.025**	0.95 <!--Soft-enter Run-on-->(0.88-1.02)	0.137	0.55 <!--Soft-enter Run-on-->(0.28-1.06)	0.076	1.28 <!--Soft-enter Run-on-->(1.00-1.64)	0.050	1.01 <!--Soft-enter Run-on-->(0.03-30.51)	0.997

<!--Col Count:19-->						Difference from Control[2]											
		Intervention		Control													
<!--Soft-enter replaced as Paramark--> 7-day PPA[1]	Time <!--Soft-enter replaced as Paramark--> Points	N	*n* <!--Soft-enter Run-on-->(%)	N	*n* <!--Soft-enter Run-on-->(%)	OR <!--Soft-enter Run-on-->(95% CI)	*p*-value	aOR[†] <!--Soft-enter replaced as Paramark--> (95% CI)<!--Soft-enter replaced as Paramark--> Ethnicity	*p*-value	aOR[b] <!--Soft-enter replaced as Paramark--> (95% CI)<!--Soft-enter replaced as Paramark--> Age	*p*-value	aOR[c] <!--Soft-enter replaced as Paramark--> (95% CI)<!--Soft-enter replaced as Paramark--> Monthly Income	*p*-value	aOR[d] <!--Soft-enter replaced as Paramark--> (95% CI)<!--Soft-enter replaced as Paramark--> Age Started Smoking	*p*-value	aOR[e] <!--Soft-enter replaced as Paramark--> (95% CI)<!--Soft-enter replaced as Paramark--> Quit Attempt	*p*-value
					>(9.4)	50.18)											
	60-day	46	30 <!--Soft-enter Run-on-->(65.2)	43	7 <!--Soft-enter Run-on-->(16.3)	6.34 <!--Soft-enter Run-on-->(0.79-50.80)	0.082	3.38 <!--Soft-enter Run-on-->(1.13-10.09)	**0.029**	0.97 <!--Soft-enter Run-on-->(0.99-1.05)	0.442	0.58 <!--Soft-enter Run-on-->(0.30-1.11)	0.097	1.33 <!--Soft-enter Run-on-->(1.02-1.73)	**0.037**	0.23 <!--Soft-enter Run-on-->(0.01-7.42)	0.408
	90-day	44	26 <!--Soft-enter Run-on-->(59.1)	43	11 <!--Soft-enter Run-on-->(25.6)	2.80 <!--Soft-enter Run-on-->(0.36-21.12)	0.327	3.72 <!--Soft-enter Run-on-->(1.24-11.10)	**0.019**	0.94 <!--Soft-enter Run-on-->(0.88-1.01)	0.103	0.59 <!--Soft-enter Run-on-->(0.29-1.22)	0.154	1.28 <!--Soft-enter Run-on-->(0.98-1.66)	0.069	0.31 <!--Soft-enter Run-on-->(0.01-9.83)	0.506
	30-day	76	24 <!--Soft-enter Run-on-->(31.6)	76	5 <!--Soft-enter Run-on-->(6.6)	4.31 <!--Soft-enter Run-on-->(0.90-20.62)	0.067	1.69 <!--Soft-enter Run-on-->(0.75-3.79)	0.204	0.94 <!--Soft-enter Run-on-->(0.87-1.01)	0.094	0.62 <!--Soft-enter Run-on-->(0.23-1.67)	0.347	1.13 <!--Soft-enter Run-on-->(0.85-1.49)	0.403	1.07 <!--Soft-enter Run-on-->(0.03-35.10)	0.971
**ITT**	60-day	76	29 <!--Soft-enter Run-on-->(38.2)	76	7 <!--Soft-enter Run-on-->(9.2)	4.00 <!--Soft-enter Run-on-->(0.77-20.82)	0.100	1.74 <!--Soft-enter Run-on-->(0.78-3.90)	0.179	0.96 <!--Soft-enter Run-on-->(0.89-1.04)	0.276	0.55 <!--Soft-enter Run-on-->(0.20-1.49)	0.239	1.15 <!--Soft-enter Run-on-->(0.86-1.54)	0.349	0.39 <!--Soft-enter Run-on-->(0.01-14.65)	0.608
	90-day	76	26 <!--Soft-enter Run-on-->(34.2)	76	11 <!--Soft-enter Run-on-->(14.5)	2.02 <!--Soft-enter Run-on-->(0.37-11.14)	0.419	1.89 <!--Soft-enter Run-on-->(0.82-4.32)	0.134	0.94 <!--Soft-enter Run-on-->(0.87-1.01)	0.110	0.57 <!--Soft-enter Run-on-->(0.18-1.80)	0.340	1.13 <!--Soft-enter Run-on-->(0.84-1.50)	0.420	0.43 <!--Soft-enter Run-on-->(0.01-15.55)	0.644
OR, odds ratio; aOR, adjusted odds ratio; CI, confidence intervals																	
[1]Measured by a self-report of continuous abstinence from smoking in the previous 7 consecutive days																	

<!--Col Count:19-->						Difference from Control[2]											
		Intervention		Control													
<!--Soft-enter replaced as Paramark--> 7-day PPA[1]	Time <!--Soft-enter replaced as Paramark--> Points	N	*n* <!--Soft-enter Run-on-->(%)	N	*n* <!--Soft-enter Run-on-->(%)	OR <!--Soft-enter Run-on-->(95% CI)	*p*-value	aOR[†] <!--Soft-enter replaced as Paramark--> (95% CI)<!--Soft-enter replaced as Paramark--> Ethnicity	*p*-value	aOR[b] <!--Soft-enter replaced as Paramark--> (95% CI)<!--Soft-enter replaced as Paramark--> Age	*p*-value	aOR[c] <!--Soft-enter replaced as Paramark--> (95% CI)<!--Soft-enter replaced as Paramark--> Monthly Income	*p*-value	aOR[d] <!--Soft-enter replaced as Paramark--> (95% CI)<!--Soft-enter replaced as Paramark--> Age Started Smoking	*p*-value	aOR[e] <!--Soft-enter replaced as Paramark--> (95% CI)<!--Soft-enter replaced as Paramark--> Quit Attempt	*p*-value
[2] Time*Group Interactions using Generalized Estimating Equations Analysis with Control Group as reference																	
[†]aOR Time + Group + Time*Group + Ethnicity																	
[b]aOR Time + Group + Time*Group + Age																	
[c]aOR Time + Group + Time*Group + Monthly Income																	
[d]aOR Time + Group + Time*Group + Age Started Smoking																	
[e]aOR Time + Group + Time*Group + Quit Attempt																	
